# Constructing a domain-specific sentiment lexicon for agricultural product reviews using BERT and SO-PMI

**DOI:** 10.1371/journal.pone.0326602

**Published:** 2025-06-26

**Authors:** Jinghua Wu, Peng Qiu, Xun Jia

**Affiliations:** College of Information Engineering, Sichuan Agricultural University, Sichuan Ya’an, China; The University of Lahore, PAKISTAN

## Abstract

The absence of a sentiment lexicon tailored to agricultural product reviews presents significant challenges for accurate sentiment analysis in this domain. Existing general-purpose lexicons, such as NTUSD, HOWNET, and BosonNLP, fail to capture the unique linguistic features of agricultural reviews, leading to suboptimal classification performance. To address this gap, this study constructs the BSTS sentiment lexicon, using a dataset of 19,843 preprocessed reviews from JD.com. Positive and negative seed words were extracted through BERT-based Term Frequency (TF) analysis, and the SO-PMI algorithm was applied to calculate sentiment scores for candidate words. By determining an optimal threshold, a balanced and effective lexicon was developed. Experimental results demonstrate that the BSTS lexicon outperforms existing lexicons in sentiment classification, achieving precision, recall, and F1 scores of 85.21%, 91.92%, and 88.44%, respectively. Furthermore, additional experiments on Taobao’s agricultural product reviews confirmed the lexicon’s robustness, with performance metrics of 93.28% precision and 87.34% F1 score, highlighting its effectiveness across different e-commerce platforms. The BSTS lexicon significantly improves sentiment classification in the agricultural domain, offering a reliable and domain-specific tool for sentiment analysis in agricultural product reviews.

## Introduction

The rapid development of e-commerce platforms has revolutionized the agricultural product market [1]. By enabling consumers to provide feedback in the form of online reviews, these platforms generate extensive user-generated data that can be mined to uncover patterns in purchasing behavior [2]. These reviews contain valuable insights into consumer satisfaction, product quality, and market trends [3]. Sentiment analysis of this wealth of review data benefits both consumers and vendors: shoppers obtain authentic insights into product performance without financial commitment, while sellers identify product shortcomings and optimize offerings accordingly [4,5]. Consequently, sentiment analysis of agricultural product reviews has become a focal point of current research.

Contemporary sentiment analysis methods fall into two main categories: deep learning–based approaches and lexicon‐based approaches. Deep learning models often achieve high accuracy but rely on large volumes of annotated training data—a costly and labor‐intensive requirement [6]. Furthermore, their “black‐box” nature limits interpretability, making it difficult to explain how specific features drive sentiment predictions [7]. In contrast, lexicon‐based methods calculate a sentiment score for each review by matching terms against a predefined emotion dictionary, then classify texts as positive or negative based on aggregate scores. There are already several existing general-purpose Chinese sentiment lexicons, including notable ones such as the Hownet Sentiment Lexicon by CNKI [8], the Chinese Positive and Negative Word Lexicon by Li Jun from Tsinghua University [9], the NTUSD Simplified Chinese Sentiment Lexicon by National Taiwan University [10], and the BosonNLP Sentiment Lexicon [11]. However, early data‐driven studies focused primarily on surface‐level indicators—such as review counts, star ratings, or average scores—thereby overlooking the rich insights embedded in review content and failing to capture deeper consumer feedback [12]. Additionally, general‐domain sentiment lexicons often cannot accommodate the specialized vocabulary and context‐dependent polarities found in agricultural product reviews [6]. For example, terms like “hard” or “organic” may carry positive connotations in one context and negative ones in another. Empirical evidence underscores the necessity of constructing domain‐specific sentiment lexicons, since a word’s sentiment orientation can vary significantly across fields [13].

Recent advancements in deep learning, such as BERT, have demonstrated exceptional capabilities in understanding contextual semantics [14]. However, the application of BERT in constructing sentiment lexicons remains underexplored, particularly in the agricultural domain [15]. Traditional lexicon construction methods often rely on generic sentiment words as seed terms, which may be less accurate for agricultural reviews. Furthermore, these methods fail to fully leverage the rich semantic embeddings provided by pre-trained models like BERT. Huang et al.[16] introduced the PTSM (Aggregated Topic Sentiment Model) for online reviews, extracting sentiment features via a lexicon and achieving strong performance with support vector machines. Nonetheless, they did not integrate deep‐learning classifiers or leverage pretrained embeddings to refine their lexicon, despite noting that lexicon‐based methods can help mitigate overfitting and filter noisy features.

Building on these findings, this paper proposes a novel sentiment lexicon construction method tailored for the agricultural product domain, referred to as BSTS(BERT-enhanced Sentiment Lexicon with TF and SO-PMI). This method enhances the adaptability of sentiment lexicons to the domain and improves the accuracy of sentiment detection in agricultural reviews. First, we fine‐tune BERT on a manually annotated corpus to obtain optimal model weights, which we then use to generate pseudo‐labels across a larger unlabeled dataset. Next, we apply Jieba tokenization and compute term‐frequency (TF) values to rank candidate “positive” and “negative” terms, selecting the top 200 seeds for each polarity. We then integrate a second‐order pointwise mutual information (SO‐PMI) algorithm, empirically determining a 15% threshold to finalize the domain‐specific lexicon. Finally, we evaluate the lexicon’s effectiveness by performing sentiment classification on held‐out test data and benchmarking our results against existing general‐domain lexicons.

The proposed domain-specific sentiment lexicon construction method has broad applications due to its minimal reliance on specific corpora. By integrating deep learning and statistical algorithms, it provides a novel approach to lexicon construction. Moreover, this study explores the potential of BERT in generating domain-specific sentiment lexicons, offering insights for sentiment analysis, customer satisfaction improvement, product optimization, and decision-making in the agricultural sector.

The remainder of this paper is organized as follows: the Related Work section discusses previous studies, the Proposed Method section introduces our approach, the Experiments and Discussion section presents our findings, and the Conclusion section summarizes the paper.

## Related work

Sentiment lexicon construction and sentiment classification are two fundamental components of sentiment analysis, particularly in their integration with deep learning models. Traditional lexicon-based approaches classify sentiment by aggregating information from sentiment-bearing words, negations, and degree adverbs within a given text [17]. While these methods are computationally efficient and widely adopted [18], their performance is heavily dependent on the quality and coverage of the sentiment lexicon.Manually curated lexicons, while offering high cross‐domain classification accuracy, demand substantial time and labor to develop [19]. **Table 1** lists the sentiment lexicons commonly used in contemporary Chinese sentiment analysis research. In the context of Chinese sentiment analysis, the challenges posed by polysemy and contextual dependencies underscore the necessity for domain-specific lexicons [20].

**Table 1:** Commonly used sentiment lexicons.

Sentiment Lexicon Name	Positive Words	Negative Words
NTUSD [2]	2812	8276
Tsinghua University’s Li Jun Chinese Sentiment Lexicon [21]	5568	4470
HOWNET [8]	4572	4574
BosonNLP [12]	10190	13711

Existing sentiment lexicon construction methods can be broadly categorized into knowledge-based expansion and corpus-based approaches. Knowledge-based expansion methods rely on a predefined set of seed words annotated with sentiment polarity, leveraging lexical relationships such as synonymy, antonymy, hierarchical structures, and semantic associations to infer the sentiment orientation of new words. Dragut et al.[22] introduced an automatic sentiment‐lexicon expansion technique to resolve the inconsistent polarity of identical terms across different dictionaries, whereas Hassan et al.[23] introduced a random walk model that determines sentiment polarity by computing transition steps between candidate words and annotated seed words. However, these methods are highly dependent on the completeness of the lexical database, limiting their scalability.

In contrast, corpus-based approaches construct sentiment lexicons by analyzing word co-occurrence patterns, contextual constraints, and vector-space representations within large text corpora. Wang et al.[24] improved the TF-IDF algorithm to assign sentiment values to words with different parts of speech, enabling the automated development of domain-specific sentiment lexicons. Ren et al.[25] employed the lightweight gradient boosting framework LightGBM to select candidate terms and utilized a pointwise mutual information (PMI) algorithm to determine their sentiment polarity, thereby constructing a domain‐specific sentiment lexicon for the electric vehicle disassembly domain. Despite their advantages, corpus-based methods struggle with determining the sentiment polarity of low-frequency words, potentially compromising lexicon completeness.

Early lexicon-based approaches often relied on static rule-based models, such as summing word sentiment scores to determine sentence-level polarity [26]. However, these methods proved inadequate for handling complex semantics, particularly in the presence of negations and modifying words. To address these limitations, hybrid approaches emerged, such as Mudinas et al.’s combination of lexicon-based tagging with Support Vector Machines (SVM), which improved sentiment classification performance [27]. Similarly, Rezaeinia et al.[28] introduced Lexicon2Vec, which integrates multiple sentiment lexicons with word embeddings to enhance semantic representation.

Recent research has explored the integration of sentiment lexicons with deep learning models to mitigate traditional limitations. For example, lexicon-enhanced Naïve Bayes models achieved over 80% accuracy in sentiment classification of medical records [29], demonstrating the potential of lexicons in filtering redundant information and reducing overfitting in deep learning-based sentiment analysis. Advances in word embedding techniques, including Word2Vec [30], GloVe [31], and FastText [32], have further enriched sentiment representation. However, even with the advent of Transformer-based models such as BERT, challenges remain in accurately capturing sentiment nuances in domain-specific contexts [33].

To address these limitations, hybrid approaches integrating lexicon-derived features with contextual embeddings have gained significant attention. For instance, Mutinda et al. proposed the Lexicon-guided N-gram Feature Extraction Model (LeNFEM), which combines sentiment lexicons with N-gram-based text representation [34]. Other studies have incorporated lexicon-derived sentiment scores into Transformer architectures, enhancing model interpretability while reducing overfitting in sentiment classification [17].

Building upon this foundation, we propose a novel method that integrates BERT with the SO-PMI algorithm to construct a sentiment lexicon specifically tailored for agricultural product reviews. The resulting lexicon and sentiment classification model demonstrated high accuracy, yielding comprehensive evaluation metrics and robust performance. These findings highlight the effectiveness of combining lexicon-based prior knowledge with deep learning techniques for domain-specific sentiment analysis.

## Proposed method

**Fig 1:**
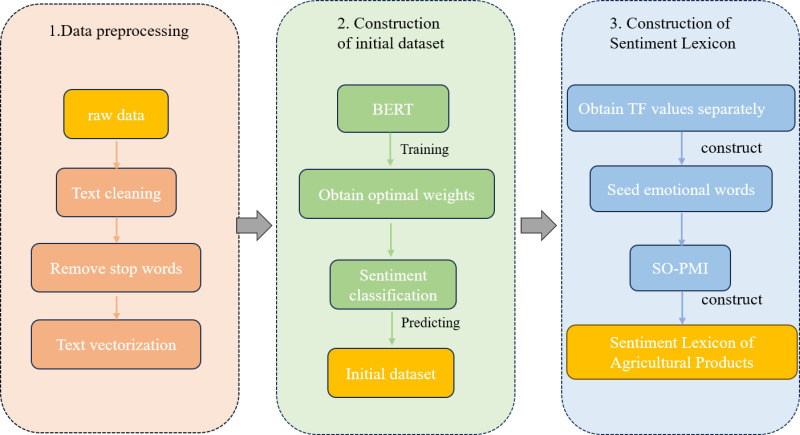
The proposed workflow for constructing the agricultural sentiment lexicon.

In this study, we propose a comprehensive sentiment analysis framework tailored for the classification of agricultural product reviews. The framework involves generating a sentiment lexicon specific to agricultural products and detailing the development steps for constructing this lexicon. **Fig 1**. illustrates the workflow for constructing the agricultural sentiment lexicon.

The algorithm for this workflow is presented in **Algorithm 1**.

Algorithm 1

**Inputs:**

  D={R1,R2,...,Rn}: Review dataset with n reviews (containing positive and negative sentiment labels).

  TF: Term frequency of words obtained using Jieba tokenizer.

  BERT: Pre-trained BERT model for fine-tuning and pseudo-label generation.

  Threshold: The SO-PMI threshold for selecting sentiment words.

**Outputs:**

  L={Lpos, Lneg}: Agricultural Product sentiment lexicon containing positive (Lpos) and negative (Lneg) words.

**Steps:**

  **Start**

  **Data Preparation:**

   Split the manually labeled dataset D (positive and negative reviews, 2000 samples total) into an 80:20 ratio for training and testing.

  **Model Training:**

   Fine-tune the BERT model on the training data using grid search to optimize hyperparameters.

   Save the best model weights.

  **Pseudo-Label Generation:**

   Use the fine-tuned BERT model to predict sentiment labels for unlabeled reviews.

   Append pseudo-labeled reviews to the training dataset.

  **Tokenization and TF Extraction:**

   Tokenize all reviews in D using Jieba tokenizer.

   Calculate term frequency TF for each token.

   Select the top 200 tokens with the highest TF values for positive and negative reviews as seed sentiment words.

  **SO-PMI Calculation:**

   For each token w:

    Compute the SO-PMI score using the formula:

      SO-PMI(w)=PMI(w,positive)−PMI(w,negative)

    Where:

      PMI(w,sentiment)=logP(w,sentiment)/P(w)*P(sentiment)

    If SO-PMI(w)>Threshold, add w to Lpos.

    If SO-PMI(w)<−Threshold, add w to Lneg.

  **Evaluate the Lexicon:**

   Use the constructed lexicon L to classify the test reviews.

   Compute evaluation metrics (e.g., precision, recall, F1-score).

  **End**

**Output:**

 Return the constructed Agricultural Product sentiment lexicon L.

In this study, we propose a domain‐adaptive sentiment lexicon construction algorithm for agricultural product reviews by combining a BERT model with SO‐PMI statistics. First, manually annotated positive and negative reviews are split into training and test sets at an 80:20 ratio. A pretrained BERT model is then fine‐tuned via grid search, and the optimal model weights are retained. Next, the fine‐tuned BERT model generates pseudo‐labels for unlabeled reviews to augment the training corpus. We apply Jieba for word segmentation across all reviews and compute term frequencies, selecting the most frequent terms from positive and negative subsets as seed sentiment words. For each candidate term w, we calculate its sentiment orientation by measuring the difference in second‐order pointwise mutual information, SO‐PMI(w), between w and the positive and negative seed sets. Terms with SO‐PMI(w) exceeding a predefined threshold are assigned to the positive lexicon; the remainder are assigned to the negative lexicon. Finally, we classify sentiment on the test set using the constructed lexicon and evaluate its performance with accuracy, recall, and F1‐score metrics, yielding the finalized positive and negative sentiment lexicons L.

### Data collection and preprocessing

#### Data collection.

This study collected online reviews of agricultural products (corn) from JD.com. The extracted data included critical fields such as review text, star ratings, review IDs, and timestamps. Review IDs were used to identify and remove duplicate entries from the same user. The primary focus of processing was on the review text, which served as the basis for identifying sentiment-bearing words to construct the sentiment lexicon. Examples of product reviews and their corresponding sentiment tendencies are shown in **Table 2**.

**Table 2:** Sample product reviews and their emotional tendencies.

Comment Text	Emotional tendencies
Sweet and glutinous white corn, with a good taste and deliciousness.	Positive
The price is too expensive, the corn is too small and too hard.	Negative

#### Data preprocessing.

**Fig 2:**
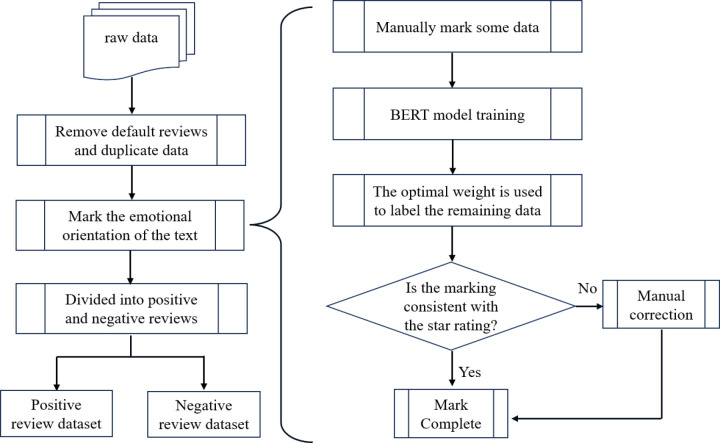
Comment preprocessing workflow.

Upon organizing the experimental data, we identified instances of noisy data, including default comments, strings containing only special characters, and duplicate entries. Additionally, we found inconsistencies between the sentiment expressed in review text and the associated star ratings, making the latter unreliable as sentiment indicators. To ensure accurate sentiment labeling, we adopted a hybrid approach combining manual annotation and machine labeling for sentiment correction. Rigorous preprocessing steps were applied to produce high-quality datasets, ensuring reliable experimental outcomes. The detailed preprocessing workflow is illustrated in **Fig 2**.

During data cleaning, default reviews with no substantive content (e.g., “This user did not leave a review”) were removed, as were reviews containing special characters or nonsensical content. Duplicate entries were also eliminated, leaving only valid text samples. To address inconsistencies between star ratings and sentiment tendencies, 2,000 reviews were randomly selected from the cleaned dataset for manual annotation. Sentiment tendencies were classified as either positive or negative. The manually labeled data were used to train and optimize the BERT-WWM model through multiple iterations, and the version with the best performance was retained. The optimal hyperparameters are shown in **Table 3**.

**Table 3:** Hyperparameter settings for the optimized BERT-WWM model.

HyperParameter	Value
Attention heads	12
Batch size	8
Epochs	3
Hidden size	768
Hidden layers	12
Learning rate	0.00005
Maximum sequence length	512
Parameters	110 M

The trained model was subsequently applied to label the remaining dataset. For reviews where the model’s predicted sentiment conflicted with the star ratings, manual validation was conducted to ensure labeling accuracy. Following this process, the dataset was divided into two subsets: one containing 9,860 positive reviews and the other 9,983 negative reviews. This preprocessing phase established a robust foundation for constructing the sentiment lexicon and conducting subsequent experiments.

### Sentiment lexicon construction

The sentiment tendencies of words in agricultural product reviews often diverge from their usage in other contexts, necessitating a domain-specific sentiment lexicon. Using the datasets generated during preprocessing, we segmented the texts using the **jieba** tokenizer. To enhance segmentation accuracy, custom dictionaries were augmented with domain-specific terms such as “Shiyue Dadian” and “Zhuanghe,” while the stopword list was expanded to include irrelevant terms like product and store names. Term frequency (TF) values were then calculated for each dataset, and the 50 most sentiment-representative words from each dataset were selected. Words reflecting positive sentiment were labeled as **pos**, and those reflecting negative sentiment were labeled as **neg**, forming a seed sentiment lexicon comprising 100 words. Examples of seed sentiment words are presented in **Table 4**.

**Table 4:** Examples of seed sentiment words.

Emotional tendencies	Seed emotional words
Positive	yummy, glutinous, satisfied, careful, sweet, high-quality, inexpensive, healthy, etc.
Negative	bad, too small, moldy, disappointment, throw, not worth it, expensive, dry, rotten, etc.

Before calculating the similarity between candidate words and the seed sentiment lexicon, part-of-speech tagging was performed to filter out auxiliary words, quantifiers, numbers, and prepositions lacking emotional orientation. The remaining words formed the candidate word set. Using the SO-PMI algorithm, we computed the pointwise mutual information (PMI) between candidate words and the positive and negative seed words. Through iterative calculations, candidates with SO-PMI values exceeding a predefined threshold were classified into positive or negative sentiment categories. This process yielded a refined agricultural sentiment lexicon with robust polarity assignments.

All study materials, data, and data analysis code can be found online (at https://osf.io/nd59y/). We hereby confirm that the extraction, processing, and analysis of customer review data from JD.com were carried out in full compliance with JD.com’s Terms & Conditions. All data were accessed in a non-commercial context, with strict adherence to the platform’s access restrictions (including its robots.txt directives) and without any unauthorized dissemination of personal information.

## Results and discussion

### Experimental data

A total of 22,531 product reviews related to agricultural products were collected from JD.com. After preprocessing and cleaning, 19,843 valid reviews remained, including 9,860 positive reviews and 9,983 negative reviews. Sample sentences from the dataset are shown in **Table 5**, illustrating the sentiment and corresponding emotional tendencies.

**Table 5:** Partial experimental data.

Example sentence	Sentiment Word	Emotional tendencies
JD.com offers affordable and trustworthy prices.	affordable	Positive
Very disappointed, it was a very unsuccessful shopping experience.	Disappointed、unsuccessful	Negative

Using the BERT-TF method, the top 50 sentiment words from the positive and negative datasets were extracted as seed words. The SO-PMI algorithm was applied to calculate the association scores between candidate and seed words. The formulae are as follows:


$$PMI(w,Pwords)=\log_{2}\frac{P(w,Pwords)}{P(w)*P(Pwords)}\;$$ (1)

$$\begin{array}{c} SO-PMI(word)=\sum {\mathrm{\backslash nolimits}}_{Pword\in Pwords}PMI(word,Pwords)-\sum {\mathrm{\backslash nolimits}}_{Nword\in Nwords}PMI(word,Nwords) \end{array}$$ (2)

Words with SO-PMI values above a threshold were classified as positive, while those below were classified as negative. Different thresholds were tested, and the results are shown in **Table 6**.

**Table 6:** Comparison results of different thresholds.

Threshold (%)	Precision	Recall	F1 Score
5	82.84	93.47	87.84
10	85.21	91.92	88.44
15	87.64	88.82	88.23
20	88.77	85.21	86.95

The optimal threshold was determined to be 10%, balancing precision and recall while maximizing the F1 Score.

### Evaluation metrics

The performance of the sentiment lexicon was evaluated using three standard metrics: Precision, Recall, and F1 Score, defined as follows:


$$Precision=\frac{TP}{TP+FP}\;$$ (3)

$$Recall=\frac{TP}{TP+FN}\;$$ (4)

$$F1\;Score=\frac{2*P*R}{P+R}\;$$ (5)

Where TP (True Positive) is the number of correctly predicted positive instances, FP (False Positive) is the number of negative instances incorrectly predicted as positive, and FN (False Negative) is the number of positive instances incorrectly predicted as negative.

### Experimental results analysis

To further evaluate the performance of the proposed sentiment lexicon (BSTS) in agricultural product reviews, we conducted additional experiments comparing the classification results on positive and negative datasets using sentiment lexicons from different sources. These lexicons include NTUSD (simplified Chinese sentiment lexicon by National Taiwan University), BosonNLP sentiment lexicon, HOWNET Chinese sentiment lexicon, and Tsinghua University’s sentiment lexicon by Li Jun. The results indicate that BSTS consistently outperforms these lexicons in terms of classification precision, recall, and F1 score, as detailed in **Table 7**.

**Table 7:** Comparison of experimental results.

Lexicon Name	Precision	Recall	F1 Score
Tsinghua	76.85	83.42	80
NTUSD	76.27	74.17	75.21
Hownet	67	94	78
BosonNLP	65.47	98.14	78.54
BSTS	85.21	91.92	88.44

BSTS outperformed other lexicons in terms of precision, recall, and F1-Score. Specifically, BSTS achieved a precision of 85.21%, which was significantly higher than the other lexicons, indicating a lower false positive rate. Additionally, BSTS had a recall rate of 91.92%, second only to BosonNLP’s 98.14%. However, BosonNLP’s lower precision (65.47%) suggests a higher false positive rate, reducing its reliability in practical applications. The F1-Score, which balances precision and recall, further confirmed BSTS’s superiority with a score of 88.44%, well above other lexicons (e.g., Tsinghua’s 80, NTUSD’s 75.21, HOWNET’s 78, and BosonNLP’s 78.54).

These results clearly demonstrate that existing publicly available sentiment lexicons are not suitable for sentiment analysis of agricultural product reviews, whereas the BSTS lexicon, tailored for this domain, significantly improved the accuracy and reliability of sentiment classification.

To verify the accuracy of the constructed sentiment lexicon in classifying agricultural product reviews on other e-commerce platforms, we collected 7,763 agricultural product reviews from Taobao. After preprocessing, 3,813 were classified as positive and 1,713 as negative. The dataset was analyzed using five sentiment lexicons, and the results are presented in **Table 8**.

**Table 8:** Taobao experimental data results.

Lexicon Name	Precision	Recall	F1 Score
Tsinghua	84.92	70	76.74
NTUSD	87.32	69.89	77.64
Hownet	81	89	85
BosonNLP	79.56	96.8	87.24
BSTS	93.28	81.93	87.34

As shown in the table, the BSTS lexicon achieved superior performance in classifying agricultural product reviews on Taobao, outperforming the other four lexicons by 5.96 percentage points in precision and registering the highest F₁-score. These findings confirm that BSTS is both more accurate and more reliable for sentiment analysis in this domain. Its consistently high precision and F₁-score, together with strong performance across multiple metrics, demonstrate its robustness and applicability to agricultural product reviews.

**Fig 3:**
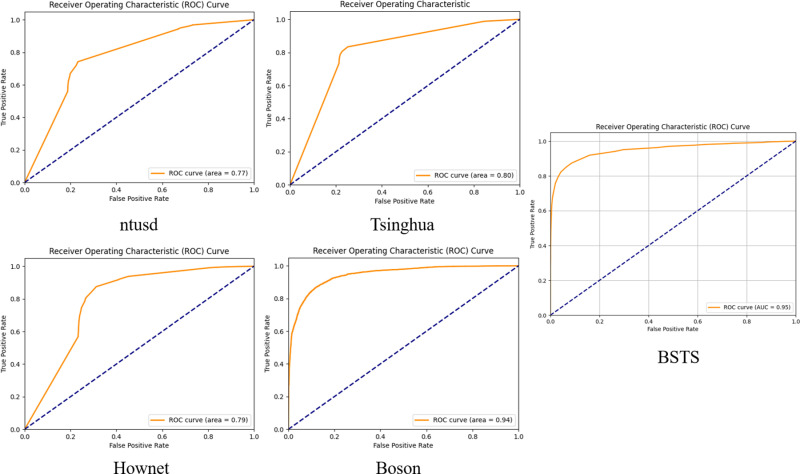
ROC mean curves of different lexicons.

**Fig 3.** illustrates the ROC (Receiver Operating Characteristic) curves for each lexicon on the agricultural product dataset. The AUC (Area Under the Curve) for BSTS exceeds those of the competing lexicons, and its ROC curve rises more steeply—evidence that BSTS more effectively captures domain-specific sentiment features. In contrast, BosonNLP, HOWNET, NTUSD, and Tsinghua’s lexicons exhibit lower AUC values and less efficient classification performance.

**Fig 4:**
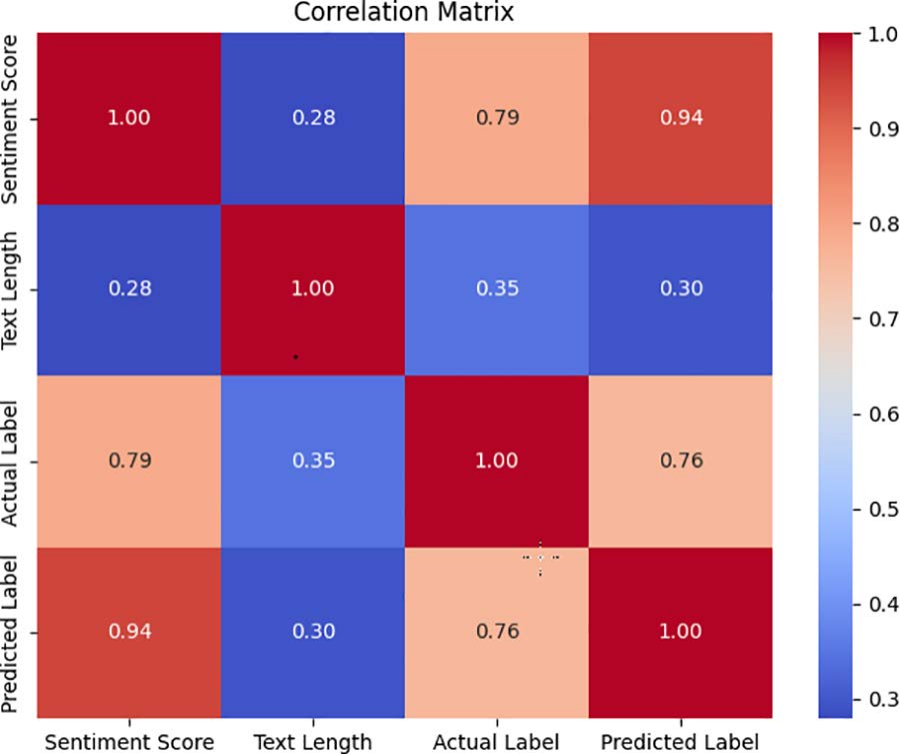
Correlation matrix.

Additionally, Fig 4 presents the correlation matrix for the BSTS lexicon. The strong correlation between sentiment scores and predicted labels (0.94) underscores the lexicon’s predictive accuracy. Conversely, the weak correlation between text length and predicted labels (0.30) indicates that text length has minimal effect on classification, further demonstrating BSTS’s robustness.

In summary, our experimental results provide compelling evidence of the BSTS lexicon’s effectiveness for sentiment analysis of agricultural product reviews. Its superior precision, recall, and F₁-score, together with consistent performance across multiple evaluation metrics, establish BSTS as a highly effective tool compared to existing sentiment lexicons.

## Conclusion

This study presents the development of a sentiment lexicon tailored to agricultural product reviews on JD.com, addressing the unique challenges of domain-specific sentiment analysis. By integrating a fine-tuned BERT model with term-frequency extraction and the SO-PMI algorithm, we developed a high-performance sentiment lexicon (BSTS) that significantly outperforms publicly available lexicons—NTUSD, BosonNLP, HOWNET, and Tsinghua—in classification tasks.

Extensive experiments on a dataset of 9,860 positive and 9,983 negative reviews from JD.com demonstrate the advantages of BSTS. It achieved a precision of 85.21%, recall of 91.92%, and F₁-score of 88.44%, surpassing all benchmark lexicons on every metric. These results underscore the limitations of general-purpose sentiment lexicons for domain-specific applications and highlight the necessity of customized lexicons to capture the specialized sentiment expressions found in agricultural product reviews.

Further validation via ROC-curve and correlation-matrix analyses confirms BSTS’s robustness. Its high AUC and steep ROC curve attest to its strong classification performance, while the low correlation between text length and prediction outcomes indicates that BSTS’s accuracy is not influenced by review length. Collectively, these findings demonstrate BSTS’s ability to deliver accurate sentiment classification without being affected by irrelevant textual attributes.

Additional experiments on Taobao’s agricultural product review dataset further confirmed the lexicon’s applicability and robustness. On this dataset, BSTS significantly outperformed the benchmark lexicons, achieving superior precision (93.28%) and F₁-score (87.34%), which underscores its reliability across different e-commerce platforms.

This research contributes to the field of sentiment analysis by offering a systematic methodology for constructing domain-specific sentiment lexicons. The integration of BERT and SO-PMI not only improves sentiment classification precision and recall but also provides a scalable approach for adapting sentiment analysis models to other domains.

Future work may explore advanced neural architectures—such as attention mechanisms—to further refine lexicon construction. Additionally, incorporating neutral sentiment annotations more effectively and conducting longitudinal studies on sentiment trends could yield deeper insights into consumer behavior and preferences.

In conclusion, the BSTS lexicon represents a significant advancement in sentiment analysis for agricultural product reviews, offering a reliable, high-performance tool for researchers and practitioners in natural language processing and agricultural information systems. Its successful application highlights the potential of combining domain-specific methodologies with advanced machine-learning techniques, paving the way for further innovations in domain-adaptive sentiment analysis.
